# Effects of Coronavirus Infections in Children

**DOI:** 10.3201/eid1602.090469

**Published:** 2010-02

**Authors:** Nicola Principi, Samantha Bosis, Susanna Esposito

**Affiliations:** University of Milan, Milan, Italy; and Fondazione Istituto Di Ricovero e Cura a Carattere Scientifico “Ospedale Maggiore Policlinico, Mangiagalli e Regine Elena,” Milan

**Keywords:** Human coronaviruses, emerging viruses, respiratory viruses, SARS, childhood, viruses, perspective

## Abstract

The isolation of the coronavirus (CoV) identified as the cause of severe acute respiratory syndrome and the detection of 2 new human CoVs (HCoV-NL63 and HCoV-HKU1) have led to studies of the epidemiology and clinical and socioeconomic effects of infections caused by all HCoVs, including those known since the late 1960s (HCoV-229E and HCoV-OC43). HCoV infections can be associated with respiratory and extrarespiratory manifestations, including central nervous system involvement. Furthermore, unlike other RNA viruses, HCoVs can easily mutate and recombine when different strains infect the same cells and give rise to a novel virus with unpredictable host ranges and pathogenicity. Thus, circulating HCoVs should be closely monitored to detect the spread of particularly virulent strains in the community at an early stage and to facilitate the development of adequate preventive and therapeutic measures.

## Abstract 2

These viruses should be closely monitored to prevent spread of virulent strains.

Human coronaviruses (HCoVs) have been known since the late 1960s as a group of viruses capable of infecting humans and animals (*1*). In a wide variety of animals, they cause respiratory, enteric, hepatic, and neurologic diseases that, in some cases (especially when they infect the young), can be severe (*1*). However, until the pathogen identified as the cause of severe acute respiratory syndrome (SARS) was isolated (*2*), the previously known HCoVs (HCoV-229E and HCoV-OC43) were considered to play a marginal clinical role in pediatrics. This conclusion was made mainly because, on the basis of the data available at the time, HCoVs were believed to cause only mild upper respiratory tract infections (URTIs) in children and that only in premature infants and children with a chronic underlying disease could severe lower respiratory tract infections (LRTIs) develop (*3*). Moreover, no importance was placed on reports that suggested a possible relationship with the development of extrarespiratory problems, including central nervous system (CNS) involvement, in which HCoVs can persist and play a role in causing chronic neurologic disorders (*4*). Consequently, the circulation of HCoVs was not monitored, and no attempt was made to develop vaccines or drugs that were active against the viruses.

The identification of SARS-CoV and the isolation of 2 novel HCoVs in humans (HCoV-NL63 and HCoV-HKU1) (*5*,*6*) have led to several studies of the epidemiology and clinical and socioeconomic effects of HCoV infections, which were greatly facilitated by the availability of modern molecular biology methods that enable direct viral identification in respiratory secretions (*7*–*26*). Interest was strengthened by the demonstration that SARS could be considered a zoonotic infection because, after it was described and the causative agent identified from patients in the People’s Republic of China, SARS-CoV–like viruses were isolated from caged animals, including palm civets and raccoon dogs in wildlife markets of the same Chinese province (*27*). This finding and the subsequent independent discovery of SARS-CoV–like viruses in horseshoe bats indicated that wild animals could be the reservoir of these viruses and that, in a suitable environment, they could infect humans and cause epidemics. New data concerning old and the new HCoVs raise the question of whether HCoVs may be more clinically important in children than was previously thought, thus indicating the need for a systematic evaluation of their circulation and the availability of preventive and therapeutic measures.

## Epidemiology of HCoV Infections in Children

A profound difference exists between the epidemiology of the infections caused by SARS-CoV and that of all other HCoV infections. SARS-CoV emerged in November 2002 and disappeared in April 2004 (*28*). During these 18 months, it was isolated in many countries, some of which were very distant from each other. However, the total of 8,098 cases of SARS diagnosed worldwide (*28*) is substantially fewer than the number usually found during epidemics of the most common respiratory viruses, such as respiratory syncytial virus (RSV) and influenza viruses (*18*).

Furthermore, seroepidemiologic studies of high-risk and low-risk residential areas have clearly shown that the prevalence of immunoglobulin G against SARS-CoV was low in children and adults (*29*); this result indicates that SARS-CoV not only had a restricted period of circulation but also that it had limited spread. Proportionally fewer children were involved: <5% of all cases were diagnosed in patients <18 years of age (*28*). The biology of SARS and its low level of transmissibility seem to be the main reasons for the low risk for contagion in children. In most of the areas in which outbreaks occurred, healthcare workers and adult patients were mainly involved and, because they were immediately hospitalized, the risk for the infection spreading to children was greatly reduced because they are not usually allowed to visit hospitals (*28*). This hypothesis seems to be further supported by the fact that the early detection and isolation of symptomatic patients were the most important measures in controlling the SARS epidemic.

Unlike SARS-CoV, HCoV-229E, HCoV-OC43, HCoV-NL63, and HCoV-HKU1 have been in continuous circulation since their first isolation and every year cause a large number of infections that more frequently involve children than adults (*3*,*5**–**26*). In particular, the data regarding the earlier-appearing HCoVs indicate that they are distributed throughout the world and mainly circulate during the winter and early spring, with outbreaks occurring every 2–4 years (*3*). Whenever HCoVs have been sought in studies of respiratory infections in infants and children, they have been found, although generally less frequently than other respiratory viruses such as RSV and influenza (*18**,**30*). However, the real importance of HCoV-229E and HCoV-OC43 in clinical practice has not been fully defined because the collected data are often discordant. In 1974, McIntosh et al. found that the global incidence of LRTIs due to HCoV-229 and HCoV-OC43 in hospitalized children was no higher than 3.8% (*31*). Later studies have shown that when all respiratory infections are considered, the etiologic prevalence of these pathogens in pediatric patients can be significantly higher, varying from ≈5% to >30% (*7*,*15*).

An overall evaluation of the available data suggests that these differences can be attributed to differences in research methods. The infections caused by these viruses are more common from November through March; more frequently affect children <5 years of age, those examined in the community, and those without underlying risk factors; and are more often identified with serologic methods (*32*,*33*). In this regard, 2 recent surveys that used serologic methods alone found previous HCoV-229E infection in 42.9%–50.0% of children 6–12 months of age (*32*) and in 65% of those 2.5–3.5 years of age (*33*). Similarly, in 75% of the cases, children 2.5–3.5 years of age had antibodies against HCoV-NL63 (*33*). All these factors can explain why the lowest incidences of HCoV-229E and HCoV-OC43 infections are usually found when the study population includes older children or adolescents, patients with underlying severe chronic diseases or hospitalized patients, when only highly symptomatic infections are considered, and when the study is conducted during the whole year.

Moreover, what has been clearly shown is that the original HCoVs are commonly detected in childhood and frequently isolated in the nasopharyngeal secretions of children with respiratory infection. In some cases, co-infections with other respiratory viruses, mainly RSV, influenza viruses, and human metapneumovirus, have also been found (*4*,*7*–*23*). However, the real incidence of HCoV-229E and HCoV-OC43 co-infections with other respiratory pathogens has not yet been defined because only a few of the published studies were planned to identify all the main respiratory viruses.

Similar conclusions can be drawn in relation to the more recently identified HCoVs. HCoV-NL63, which can be found in 1.0%–9.3% of nasopharyngeal aspirates from patients with RTIs (*7*–*23*), circulates throughout the world (predominantly during the winter in temperate regions), infects mainly younger children and subjects with underlying severe chronic diseases, and is more frequently found in nonhospitalized children (Appendix Table). Although it was not isolated until 2000, HCoV-NL63 has probably been circulating for some time because one of the first detections occurred in a sample of nasopharyngeal secretions collected from a child with pneumonia that had been kept in a freezer since January 2003 before evaluation (*5*).

HCoV-HKU1 was identified in 2005 and has once again been found in nasopharyngeal secretions of children and adults with respiratory infections in countries that are very distant from each other. Its incidence varies from <1% to 6% (Table) (*6*,*20*,*24**–**26*), and seroepidemiologic surveys based on antibodies reacting with the recombinant HKU1 nucleocapsid protein suggest that infection may be relatively common in humans, although generally asymptomatic (*32**,**33*). HCoV-NL63 and HCoV-HKU1 are often associated with co-infections with other respiratory viruses, mainly RSV and influenza viruses (*7**–**26*).

**Table:** Main studies of the epidemiology and clinical relevance of HCoV-HKU1 in infants and children*

Study	Location and period	Population	No. samples tested	No. (%) patients with positive test results	Comments
Lau et al. (*20*)	Hong Kong; 2004 Apr–2005 Mar	629 children with RTIs, 6 mo–5 y; inpatients	629	10 (1.6)	11 patients with URTIs, 1 with pneumonia, 1 with bronchiolitis, 5 with febrile seizures; 3 with underlying disease
Vabret et al. (*24*)	Canada; 2005 Feb–Mar	83 children with RTIs, <5 y; negative for RSV, influenza A/B, PIV 1–3, adenovirus; inpatients	83	5 (6.0)	3 patients with gastroenteritis, 1 with febrile seizures; mean age 26 mo
Sloots et al. (*25*)	Australia; 2004 May–Aug	259 children with RTIs, <5 y; inpatients and outpatients	259	10 (3.8)	1 patient with co-infection
Talbot et al. (*34*)	USA; 2001 Oct–2003 Sep	1,055 children with RTIs, <5 y; inpatients	1,055	4 (0.4)	Mild episodes

*HCoV, human coronavirus; RTI, respiratory tract infection; URTI, upper respiratory tract infection; RSV, respiratory syncytial virus; PIV, parainfluenza virus.

## Clinical manifestations of HCoV infections in children

### Respiratory Problems

It is well known that all HCoVs cause respiratory infections. SARS-CoV is the most aggressive, although the disease seems to be substantially less severe in children than in adults. In patients <12 years of age, the clinical course of SARS was generally milder and shorter than in those >12 years: no death was reported, only 5% of the infected children were admitted to an intensive care unit, and <1% required mechanical ventilation (*28*). Leung and Chiu found that several children with SARS-CoV infection recovered without any sequelae after receiving supportive therapy alone (*36*). The only pediatric patients with severe respiratory problems associated with SARS-CoV infection were >12 years (*36*). The clinical picture in persons <12 years was similar to that caused by other respiratory viruses, including influenza viruses. Moreover, the extrarespiratory manifestations of SARS-CoV infection described in adults (hepatitis and CNS dysfunction) have never been reported in children.

The clinical role of all non-SARS CoVs seems to be similar: in most healthy children: they cause URTIs that spontaneously disappear in a few days. This finding is clearly shown by our data indicating no difference in the incidence and clinical severity of the diseases associated with HCoV-229E, HCoV-OC43, and HCoV-NL63. Regardless of the HCoV causing the infection, >50% of the children had a common cold or pharyngitis, and laboratory and radiologic investigations were required in <15% (*18*). Moreover, we also found that the socioeconomic effects of these viruses on the families of the infected children was marginal: the viruses spread significantly less than influenza viruses among household members, caused only a limited number of similar infections in the family, and led to fewer lost working or school days (*18*).

Although possible, the association of non-SARS HCoV infection and LRTI is uncommon in healthy children. In most published studies, the incidence of pneumonia or bronchiolitis was <5% (*7*–*26*,*34*). Differences in the incidence of LRTIs among studies can at least partially be attributed to the different prevalence of co-infections. Gerna et al. found a high incidence of HCoV-229E, HCoV-OC43, and HCoV-NL63 infections in infants and children with bronchitis, bronchiolitis, or pneumonia, but most of the LRTIs were demonstrated in children co-infected with HCoVs and other respiratory viruses (*37*). Furthermore, the method used to collect respiratory samples can also play a role in explaining the greater incidence of LRTIs (*11**,**14**,**16**,**22*). In this regard, it is important to emphasize that when only hospitalized patients (with, consequently, only the most severe cases) are enrolled, the incidence of LRTIs seems greater (*34**,**37*).

Unlike in healthy children, the development of severe clinical features after infection with non-SARS HCoV is relatively common among newborns, premature and low birthweight infants, and children at risk because of underlying health problems. Gagneur et al. described 3 HCoV-229E–related outbreaks in a pediatric and neonatal intensive care unit in France during 1998 (*38*), and 75% of the neonates and 92% of the extremely premature infants were symptomatic. Kuypers et al. studied the contribution of non-SARS HCoVs to acute RTIs and found that several children with isolated HCoV disease had an underlying medical condition (*21*).

Recently collected data concerning the individual viruses indicate that HCoV-NL63 may be more frequently associated with croup than with HCoV-229E or HCoV-OC43. Van der Hoek et al. found that 9 (45%) of 20 children infected by HCoV-NL63 alone, and 12 (25%) of 49 HCoV-NL63–positive children as a whole, had croup, compared with 54 (6%) of 900 HCoV-NL63–negative children (p<0.001) (*16*). Wu et al. (*22*) and Han et al. (*35*) also reported a high prevalence of croup in children infected with HCoV-NL63. Furthermore, other reports indicate that both HCoV-NL63 (*9**,**12**,**15**,**16**,**21**)* and HCoV-HKU1 (*20**,**26*) are associated with the development of bronchiolitis and wheezing.

### Extrarespiratory Problems

As mentioned above, SARS-CoV does not seem to cause extrarespiratory problems in children, but all of the other HCoVs can be associated with signs and symptoms involving organs and systems other than the respiratory tract. Abdominal pain, emesis, and diarrhea can be the first signs and symptoms of an acute infection due to non-SARS CoVs. These manifestations have been reported, particularly in the cases of HCoV-OC43 and HCoV-NL63, and seem to be the direct consequences of viral invasion of the intestinal mucosa, as suggested by the presence of HCoV-like particles in the stool samples of many patients with acute disease (*3*,*7*–*23*).

Non-SARS CoV infections have also been associated with acute and chronic CNS diseases (*4**,**20*), although no clear evidence has shown that the viruses played a direct causative role. Nevertheless, some evidence exists of a possible relationship between HCoV infection and CNS damage. HCoV-229E and HCoV-OC43 infections have been associated with the development of various chronic neurologic disorders, including multiple sclerosis, because these viruses have been found more frequently in the autopsied brain tissue of patients with these diseases more frequently than in healthy patients (*4*). A possibly causative role of HCoV-OC43 in determining chronic brain damage is further supported by the fact that chronic demyelinisation of mouse CNS can be induced by infection with another CoV, mouse hepatitis virus (MHV), which belongs to the same antigenic group as HCoV-OC43 and has structural similarities with it (*39*).

Because MHV induces the secretion of pro-inflammatory molecules, such as interleukin-1β (IL-1β), tumor necrosis factor, IL-6, and macrophage-inflammatory 1β, during the infection of neural cells, HCoV-OC43 may act similarly in the CNS of infected children and lead to severe brain damage. Furthermore, SARS-CoV (which has many genetic similarities to both viruses) seems to cause lung damage by activating the same pro-inflammatory molecules, because a particularly high level of circulating IL-1β has been found in children with SARS (*34*).

An association of acute neural disease with HCoV infection has been clearly demonstrated by the detection of HCoV-OC43 in the cerebrospinal fluid of a child presumed to have acute disseminated encephalomyelitis, and the frequent association between HCoV-HKU1 infection and the development of febrile seizures seems to lead to the same conclusion. Lau et al. studied 10 children infected by this virus and found that half were affected by febrile seizures, the highest prevalence among all the HCoVs (*20*). Because the fever in all of these children was not particularly high and lasted for a shorter period than fever associated with other viral respiratory infections, it was considered unlikely that all were simple febrile seizures, but possible that they may represent specific neurologic damage induced by HCoV-HKU1 or that the virus may trigger a negative immune response.

Finally, Esper et al. identified HCoV-NL63 in respiratory specimens from 8 (72.7%) of 11 children with Kawasaki disease (KD) and in only 1 (4.5%) of 22 age-matched controls, thus suggesting that KD may be triggered by a response to HCoV-NL63 infection (*40*). However, the findings of other studies do not support this observation, and so the question of the causative role of HCoV-NL63 in the development of KD remains unanswered.

## Assessment of the importance of known HCoVs in Children

Superficial analysis of all of the available data concerning the effects of HCoVs in children suggests that the assessment of the importance of HCoVs made before SARS-CoV was identified can still be considered valid. In general, all of these viruses (including SARS-CoV) have been confirmed as mainly respiratory viruses with limited clinical relevance in children. They cause mainly URTIs, are not frequently isolated in hospitalized children (*7*–*26*), and, because they are rarely transmitted to other household members, have a marginal socioeconomic effects on families (*18*). Even SARS-CoV infection, which had a dramatic effect on adults, was mainly associated with relatively mild disease in almost all patients <12 years of age (*28**,**34*). Moreover, most children with a diagnosis of severe respiratory syndrome in whom a HCoV was isolated were co-infected by other respiratory viruses (*7*–*26*). These finding suggest that the severity of the respiratory disease at least in some of these cases was attributable to the second virus.

Because only premature infants, neonates with a low birthweight, and children with an underlying severe chronic disease are at risk of experiencing a severe respiratory problem associated with HCoV infection (*21*,*37*), we could conclude that no further studies of the role of HCoVs in children are needed because what is already known is enough to make such investigations superfluous. Furthermore, on the basis of the data regarding the natural outcome of respiratory infections, developing vaccines or specific drugs appear to be unnecessary.

However, different conclusions can be drawn when the global spectrum of the diseases caused by these viruses in animals and humans is considered. It is now well known that an enormous reservoir of CoVs exists among animals, particularly horseshoe bats, and that CoV isolates recovered from animals in China have up to 99.8% nucleotide identity with SARS-CoV (*27*). Because CoVs can easily mutate, this means that (as in 2003) sustained exposure to the infected animals can lead to a SARS-like CoV strain that is newly adapted to infect humans and capable of causing the reappearance of SARS. Moreover, it has been shown experimentally and in nature that all CoVs undergo a high rate of genetic mutations and can recombine when 2 different strains infect the same cells (*1*). This finding means that it is theoretically possible that future situations similar to those involving SARS-CoV may involve CoVs that currently infect only some animals, thus leading to novel viruses with unpredictable host ranges and pathogenicity.

Phylogenetic analyses of the genes spanning the HCoV-HUK1 genome suggest that this virus may be the result of a recombination event between related but distinct HCoVs (*6*) and that SARS-CoV may have originated from a unique recombination (*2*). In this regard, the behavior of CoVs could be quite similar to that of influenza viruses, for which genetic changes and recombinations of avian or swine strains are required to allow them to cross the species barrier and replicate in humans to cause a pandemic.

Consequently, as is usually the case with influenza, a systematic evaluation of the characteristics of CoVs should be planned. Patients with severe respiratory syndrome seem to be the best target for this kind of evaluation and, in this population, studies of children (in whom the incidence of infection is higher) may also have application to adults because the findings may lead to a reduction in the risk for the spread of particularly virulent HCoV strains.

In addition to the risk for a pandemic related to the reappearance of SARS or other new CoVs, the data regarding the possible relationship between HCoV infection and CNS diseases also suggest the need for a systematic evaluation of the circulation of CoVs. If >1 HCoVs are demonstrated to play a real role in causing some of the CNS diseases with which they have been associated, substantial changes would be required in our diagnostic, prophylactic, and therapeutic approaches to many neurologic illnesses in children.

## Conclusions

HCoV infections can be associated with respiratory and extrarespiratory manifestations, including central nervous system involvement. The clinical and genetic characteristics of circulating HCoVs in the pediatric population should be monitored to detect the spread of particularly virulent HCoV strains in the community at an early stage and, if required, to facilitate the development of adequate preventive and therapeutic measures.

## Supplementary Material

Appendix Table Main studies of the epidemiology and clinical relevance of HCoV-NL63 in infants and children*
