# Supplementary material for: Effects of Coronavirus Infections in Children
Source: Emerg Infect Dis. 2010 Feb;16(2):183–8. doi: 10.3201/eid1602.090469 (PMC2957994; doi:10.3201/eid1602.090469)
Supplement: Appendix Table — Main studies of the epidemiology and clinical relevance of HCoV-NL63 in infants and children* [file 09-0469_appT-s1.pdf]

Appendix Table. Main studies of the epidemiology and clinical relevance of HCoV-NL63 in infants and children*

Study	Location and period	Population	No. samples tested	No. (%) patients with positive test results	Comments
Fouchier et al. (*7*)	The Netherlands; 2000 Nov–Jan 2002	122 children <16 y with RTIs; negative for RSV, influenza A/B, hMPV, PIV 1–4, adenovirus, rhinovirus, HCoV-229E, HCoV-OC43; inpatients	122	5 (4.1)	1 patient with pneumonia; mean age 27.8 mo (4–8 mo); 4 with underlying disease
Esper et al. (*8*)	USA; 2002 Jan–2003 Feb	895 children <5 y with RTIs; negative for RSV, influenza A/B, PIV 1–3, adenovirus; inpatients and outpatients	1,265	79 (8.8)	URTIs and LRTIs; mean age 6.5 mo;12% co-pathogens; 2 premature infants died
Suzuki et al. (*9*)	Japan; 2002 Jan–2003 Dec	419 children of all ages with RTIs; negative for RSV, influenza A/B, PIV 1–3, rhinovirus, CMV, HSV, enterovirus, measles virus, mumps virus; inpatients and outpatients	419	5 (1.2)	4 patients with URTIs and 1 patient with bronchiolitis; mean age 55 mo; 2 with predisposing factors
Chiu et al. (*10*)	Hong Kong; 2001 Aug–2002 Aug	587 children of all ages with RTIs or febrile seizures. Inpatients	587	15 (2.5)	5 patients with URTIs, 4 with febrile seizures, 2 patients with croup; mean age 30.7 mo; 5 co-infections
Arden et al. (*11*)	Australia; 2001 Nov–2004 Feb	766 adults and children (73% of the study patients <5 y); inpatients and outpatients	840	16 (2.0)	14 cases in children; 11 with LRTIs (especially bronchiolitis); mean age 15 mo
Ebihara et al. (*12*)	Japan; 2002 Oct–2003 Sep	118 children with bronchiolitis; negative for RSV, influenza A/B, hMPV; inpatients	118	3 (2.5)	Mean age 11.5 mo
Kaiser et al. (*13*)	Switzerland; 2003 Jan–2003 Dec	82 infants followed up for 1 y; first LRTI analyzed	82	6 (7.3)	Mean age 6 mo.1 co-infection
Bastien et al. (*14*)	Canada; 2002 Nov–2003 Dec	Children <17 y with RTIs; most cases negative for other respiratory viruses; inpatients and outpatients	1,240	26 (2.1)	Age 7 d–9.5 y (65.4% <1 y);1 co-infection
Vabret et al. (*15*)	France; 2002 Nov–2003 Apr	237 children <5 y with RTIs; negative for RSV, influenza A/B, rhinovirus, hMPV, HCoV-229E, HCoV-OC43; inpatients	237	22 (9.3)	6 patients with bronchiolitis, 1 patient with pneumonia
Van der Hoek et al. (*16*)	The Netherlands; 1999 Dec–2001 Oct	949 children with LRTIs, <3 y; inpatients and outpatients	949	49 (5.2)	23 patients with bronchiolitis, 12 with croup, 5 with pneumonia; mean age 0.7 y for inpatients and 1.5 y for outpatients; 29 coinfections
Boivin et al. (*17*)	Canada; 2001 Dec–2002 Apr, 2003 Jan–May	396 children with RTIs, <u><</u>3 y; inpatients	396	12 (3.0)	2 patients with URTIs, 9 with bronchiolitis or bronchitis and pneumonia; mean age 10.1 mo; 9 co-infections
Esposito *et al*. (*18*)	Italy; 2003 Nov–2004 Mar	2,060 otherwise healthy children 0–14 y attending emergency unit for acute disease, excluding trauma	2,060	20 (1.0)	3 patients with LRTIs; mean age 24 mo; 7 co-infections
Choi et al. (*19*)	Korea; 2000 Sep–2005 Aug	515 children with LRTIs, <u><</u>5 y; inpatients	515	6 (1.1)	2 patients with pneumonia, 3 with croup, 1 with asthma; mean age 15.4 mo
Lau et al. (*20*)	Hong Kong; 2004 Apr–2005 Mar	629 children with RTIs, 6 mo–5 y	629	14 (2.2)	4 patients with febrile seizures
Kuypers et al. (*21*)	USA; 2003 Oct–2004 Sep	1,043 children with RTIs, 0–19 y; outpatients	1,043	11 (1.0)	Many patients with co-infections and underlying disease
Wu et al. (*22*)	Taiwan; 2004 May–2005 Apr	539 children <15 y	539	7 (1.3)	5 patients with croup, 2 with pneumonia; 3 with co-infections

Smuts et al. (*23*)	South Africa; 2004 May–2005 Nov	238 children with acute wheezing, 2 mo–6 y; outpatients	242	6 (2.4)	Most mild episodes
Talbot et al. (*34*)	USA; 2001 Oct–2003 Sep	1,055 children with RTIs, <5 y; inpatients	1,055	12 (1.1)	Most mild episodes
Han et al. (*35*)	Korea; 2004 Apr–2006 Apr	872 children with RTIs, 0–16 y; inpatients	872	14 (1.7)	9 patients with croup, 1 patients with asthma exacerbation, 1 patient with pneumonia

*CMV, cytomegalovirus; flu, influenza; HCoV, human coronavirus; hMPV, human metapneumovirus; HSV, herpes simplex virus; LRTI, lower respiratory tract infection; PIV, parainfluenza virus; RSV, respiratory syncytial virus; RTI, respiratory tract infection; URTI, upper respiratory tract infection.
